# Description of Two Cases of Anaplastic Large Cell Lymphoma Associated with a Breast Implant

**DOI:** 10.1155/2019/6137198

**Published:** 2019-06-27

**Authors:** Julie Crèvecoeur, Véronique Jossa, Joan Somja, Jean-Claude Parmentier, Jean-Luc Nizet, André Crèvecoeur

**Affiliations:** ^1^Center of Senology Drs Crèvecoeur, Liège, Belgium; ^2^Laboratory of Anatomy and Pathology, CHC St-joseph, Liège, Belgium; ^3^Laboratory of Anatomy and Pathology, CHU, Liège, BelgiumBelgium; ^4^Department of Plastic and Maxillofacial Surgery, CHU, Liège, BelgiumBelgium

## Abstract

Breast implant-associated anaplastic large cell lymphoma (BIA-ALCL) is a recently recognized provisional entity in the 2017 revision of the World Health Organization classification of lymphoid neoplasms. Although the majority of the cases described in the literature demonstrate an effusion confined to the capsule of the breast implant, this rare pathology can also invade the capsule and adjacent tissues and/or involve lymph nodes. We hereby report two new cases of BIA-ALCL in a 58-year-old and a 47-year-old Caucasian female who received a silicone breast implant. The first patient showed a sudden and rapid right breast volume increase 6 years after the implantation surgery. As for the second patient, a left breast volume increase was observed also suddenly and quickly 11 years after surgery. In both cases, an uncompressed mammography was performed allowing a new approach to highlight periprosthetic fluid reaction. Pathologic examination of the fluid collection revealed atypical cells positive for CD30 and CD45 and negative for ALK and CK7. This allowed pathologists to diagnose a breast implant-associated anaplastic large cell lymphoma. Patients were treated with bilateral capsulectomy with no additional local or systemic therapy. The development of breast augmentation may come with an increase in the frequency of this pathology. Radiologists and senologists must therefore be careful when women with breast implants show an increase of breast volume and all cases of BIA-ALCL must be recorded and reported.

## 1. Introduction

Malignant Lymphomas of the breast are rare and account for less than 0.5% of all malignant breast tumors [1, 2]. The most common types of breast lymphomas are B-cell lymphomas with only rare cases showing T-cell phenotype [3, 4]. Anaplastic lymphoma kinase (ALK) negative anaplastic large T-cell lymphoma (ALCL) is a rare form of Non-Hodgkin's lymphoma [5]. Breast implant-associated ALK-negative ALCL (BIA-ALCL ALK negative) has been reported for the first time in 1997 by Keech and Creech [6] and 14 years passed before it was recognized as a distinct clinicopathologic entity [5]. In 2017, BIA-ALCL was identified as a provisional entity in the revised WHO 2017 classification [7]. In February 2019, the US Food and Drug Administration (FDA) analyzed 660 medical devices reports (MDRs) of BIA-ALCL and concluded that 457 MDRs meet the pathologic criteria of BIA-ALCL [8]. Some authors described two subtypes of BIA-ALCL, the* in situ* BIA-ALCL consisting of an anaplastic proliferation with “hallmark” cells confined to the capsule in association with seroma and the infiltrative BIA-ALCL where cells infiltrate the capsule and/or adjacent tissues and is associated with a tumor mass [9]. Today it seems more appropriate to consider the clinical and pathologic staging of BIA-ALCL following the TNM staging system defined by Clements et al. [10]. In this tumor staging system, T1 means that the lymphoma cells are confined to the luminal surface, T2 indicates an early capsule invasion, T3 when tumors cells invade deep into the capsule but not beyond and T4 implies the presence of a mass and tumor cells out of the capsule [10]. The treatment of patients with BIA-ALCL will be adjusted according to the stage of the disease. However, the complete surgery involving a total capsulectomy and the breast implant removal is the optimal treatment for most patients. An incomplete resection, an associated mass, or an involvement of lymph node will require an adjuvant treatment such as chemotherapy or radiation therapy [10–13]. There is no role for a radical mastectomy and sentinel node biopsy, and full axillary dissection is reserved only for multinode metastasis. The prognosis of the disease mostly depends on the extent of the disease at the time of diagnosis. Compared with many other subtypes systemic T-cell lymphoma, the prognosis of BIA-ALCL in early stage is excellent [11, 12].

In this study we report two clinical cases of BIA-ALCL found at the same institution. This case report contributes to a better understanding of this pathology and may be helpful in determining the best treatment for these patients.

## 2. Cases Presentation

### 2.1. Case 1

**Figure 1:**
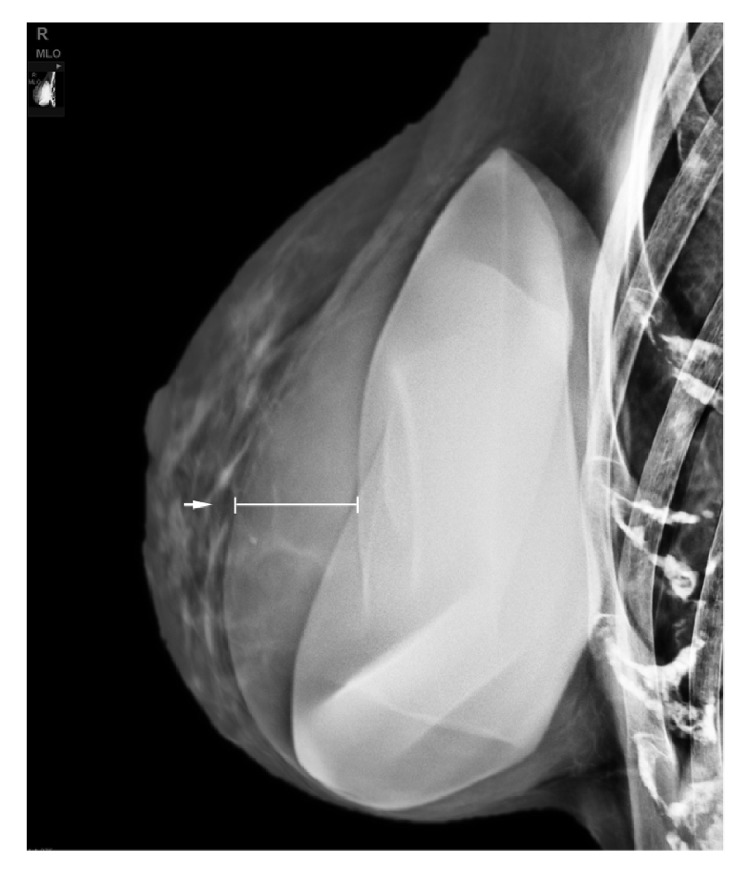
Mediolateral oblique view of the right breast shows a rim of density surrounding the silicone implant (white arrow).

**Figure 2:**
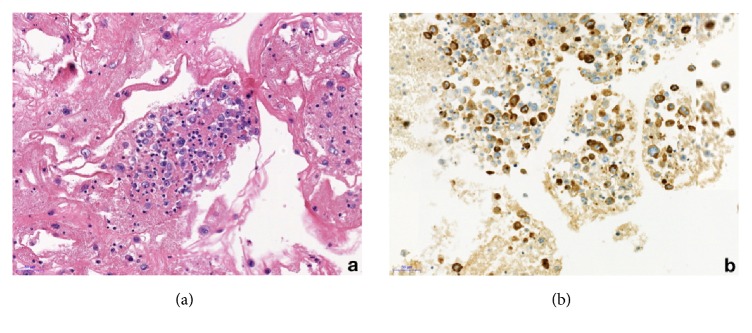
Case 1, representative section of the cell block preparation from effusion fluid. (a) A cluster of lymphoma cells is observed (hematoxylin and eosin); (b) CD30 expression by the neoplastic cells of BIA-ALCL (original magnification 200x).

A 58-year-old woman came to our Center of Senology with an increase in volume of her right breast without sign of an infection. She is the mother of four children and under IUD Mirena and estrogel treatments. Her breast surgery history is the following: in 2003, excisional biopsy of a cluster of superior right-sided calcifications corresponding to benign calcifications on focus of fibrocystic mastopathy; in 2004, breast augmentation with retropectoral silicone implants and mastopexy; in 2011, replacement of implants following intracapsular rupture of the left one. The reference of her implants is the following: Allergan Inspira TSLP with volume of 300cc and textured surface. In her hereditary history, her maternal grandmother was diagnosed with breast cancer. The clinical examination revealed that her breasts are clearly dissymmetrical. The right breast being much larger and bulging than the left one, without suspicious mass detected. There is no skin retraction, no erythema, and no palpable lymphadenopathy. When performing mammography on a patient with breast implants, the guidelines specify to use Eklund's technique [14]. This technique comes with a limitation with the breast implant not visible on the photography. Consequently, we always perform an X-ray of the entire breast without compression before applying the method. This allows us to check the integrity of the prosthesis, to locate it and to estimate the proportion of mammary glands. Our center is equipped with a senographe Essential Full-Field Digital System from General Electric (GE) medical system company. On the mammography, the mediolateral oblique (MLO) view without compression demonstrates that the right prosthesis is deformed on its anterior surface (Figure 1). In addition, there is a predominantly periprosthetic effusion on the anterior surface, which explains this deformation of the prosthesis. The effusion seems located in the prosthetic capsule. In Eklund incidence, no lesion has been observed. We performed a bilateral ultrasound with Canon Aplio i600 from Canon medical system company. This exam does not show any tumoral lesion or cyst. But it confirms the existence of a significant right periprosthetic fluid reaction. To obtain a diagnosis, a partial evacuation by fine needle aspiration was performed and 100cl of yellowish liquid was removed. The bacteriologic analysis of a part of this sample demonstrates no sign of infection. The pathologic evaluation of the second part of this sample firstly reveals the presence of atypical cells (Figure 2(a)). Immunohistochemical analysis then demonstrated a moderate and diffused expression of CD30 (Figure 2(b)). Moreover CD45 and Vimentins are expressed while ALK and CK7 are not. This sample was submitted for a blind analysis to four independent anatomopathologists who all came to the conclusion of a breast implant-associated anaplastic large cell lymphoma. The effusion is confined between the capsule and the prosthesis without extension beyond the capsule of the implant and no mass or lymph node is detected. Thus, the BIA-ALCL-specific TNM staging system designed by Clements et al. [10] for this patient is T_1_N_0_M_0_ (Stage IA). The optimal treatment for patients with this stage is a complete surgical excision of the prosthesis and the capsule [10–13]. Consequently, our patient underwent bilateral capsulectomy and the right and left periprosthetic capsules were analyzed. Both capsules were entirely sampled and one section of each block was analyzed with particular attention to the luminal side. The histological and immunohistochemical analysis of the periprosthetic capsule did not show lymphomatous infiltration. A CD30 immunohistochemistry performed on each slide was negative. Three weeks after the surgery, the patient underwent a PET/CT scan which showed a discrete parieto-thoracic bilateral hyperfixation with a slightly more intense signal at the level of the 3^rd^ right chondrocostal junction. However, this signal is unspecific given the postsurgical context. The disease was localized and no standard approach for systemic treatment for this patient was recommended [12]. Now, the patient has been disease-free with no evidence of disease recurrence for two years.

### 2.2. Case 2

**Figure 3:**
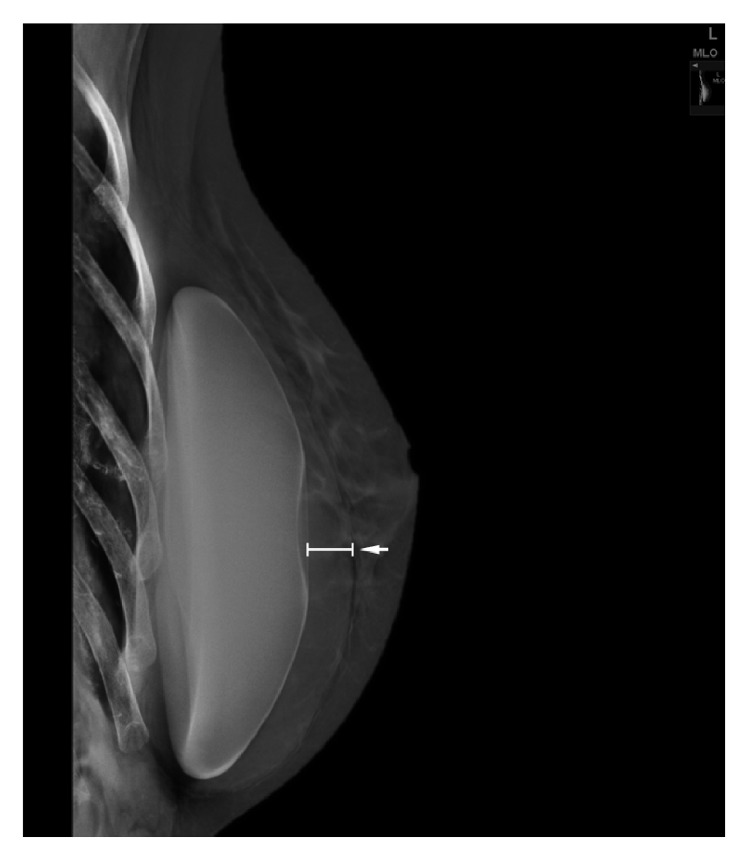
Mediolateral oblique view of the left breast shows a rim of density surrounding the silicone implant (white arrow).

**Figure 4:**
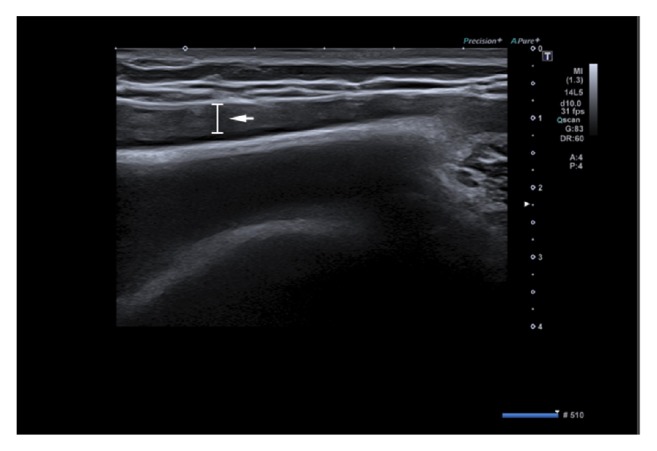
Ultrasound of the left breast demonstrates periprosthetic fluid reaction in the inner region (white arrow).

**Figure 5:**
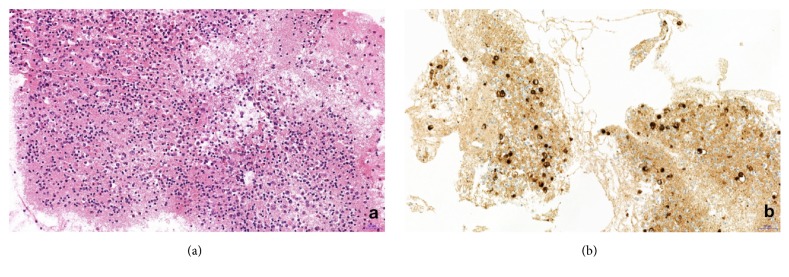
Case 2, (a) H&E section of the cell block preparation from effusion fluid showing atypical lymphocytes; (b) CD30 immunohistochemistry highlights tumor cells positive for CD30 (original magnification 200x).

A 47-year-old woman came to our center with an increase in volume of her left breast without sign of infection. She is the mother of two children, without hormonal treatment. She has no hereditary history of breast cancer. In 2004, a breast augmentation was performed using retropectoral textured silicone gel implants, Allergan style 110, 330cc. The clinical examination revealed that her breasts were slightly dissymmetrical. The left breast was much larger than the right without suspicious mass detected and no palpable lymphadenopathy. On the mammography, the mediolateral oblique (MLO) view without compression demonstrates that the left prosthesis is deformed on its anterior pole and a periprosthetic collection developed mainly on the anterior surface of the prosthesis (Figure 3). In Eklund incidence, no lesion is observed. We then performed a bilateral ultrasound. This exam did not show any tumoral lesion or cyst in both breasts. But it confirmed the existence of a significant left periprosthetic fluid reaction predominant in the inner region (Figure 4). To get a diagnosis, a partial evacuation by fine needle aspiration is performed. The bacteriologic analysis of a part of this sample demonstrated no infectious sign. The pathologic evaluation of the second part of this sample identified atypical cells (Figure 5(a)) and a positivity for CD30 (Figure 5(b)). Immunohistochemical analysis demonstrated an expression of CD45 and CD3. The cells did not express ALK and CK7. This sample was also submitted for a blind analysis to four independent anatomopathologists who all came to the conclusion of a breast implant-associated anaplastic large cell lymphoma. For this patient, the BIA-ALCL-specific TNM staging system is T_1_N_0_M_0_ (Stage IA). The patient underwent bilateral implant removal and capsulectomy. The total samples were analyzed. Similarly to the first case, no lymphomatous infiltration was found in the periprosthetic capsule and CD30 remained negative on each slides. Fifteen days after the surgery the patient received a PET/CT scan which showed a slight hypermetabolic area in the left side but unspecific. No other metabolic lesion was found. Considering the stage of the disease and the complete surgical excision including total capsulectomy and the absence of lymphomatous infiltration, no additional treatment was performed. The patient remains clinically well after 24 months of followup under close surveillance by our center and hematology clinic.

## 3. Discussion

In this study, we described two case reports of BIA-ALCL, consisting of an anaplastic proliferation with “hallmark” cells confined to the capsule associated with a seroma. Contrary to the other studies, the diagnosis was confirmed before performing the capsulectomy. Indeed, the mammographies without compression showed clearly a periprosthetic effusion. Moreover, ultrasound imaging is one of the most sensitive techniques for the detection of peri-implant fluid collection and has the added benefit of being readily available for ultrasound image guided aspiration and diagnosis [15, 16]. The histological and immunohistochemical analyses and consequently the diagnosis of BIA-ALCL were established from the fine needle aspiration performed by the senologist. The first treatment for BIA-ALCL is the removal of the implant and the resection of the entire capsule [12, 17–19]. In the cases presented here, the anatomopathological analysis of the periprosthetic capsule, following the capsulectomy, demonstrated the absence of lymphomatous infiltration. But that does not call into doubt the diagnostic of BIA-ALCL proven with the needle aspiration. The drainage of the effusion may indeed lead to reaccumulation of the fluid in a few weeks but the number of lymphoma cells may be considerably decreased by dilution and neutrophils or histiocytes are generally observed [13]. Laurent et al. demonstrated in a retrospective study that complete capsulectomy and implant removal is required for all cases of BIA-ALCL and adjuvant therapy is considered according to the subtype [9]. An incomplete resection or positive margins or advanced disease require chemotherapy and chest wall radiation. Anti-CD30 immunotherapy and stem cell transplant are actually under investigation [17].

Previous studies reported different incidences of developing ALCL in women with breast implants [19, 20] and the most recent report by Clemens M. in FDA estimated the risk between 1 per 3817 to 1 per 30 000 women with textured implant [8]. Thus, it seems important to report in the literature every case of BIA-ALCL and determine if similarities are observed between the different cases. The published data have already produced interesting findings. One of the common factors for the cases reported in the literature is the type of implant. In our two cases, the implant surface is textured. Many studies have already reported a possible link between the implant surface and the BIA-ALCL pathology. Indeed, it is possible that, with a textured surface in particular, an immunologic response could develop due to chronic irritation by the prosthesis [9, 21]. Some authors have suggested that the trigger for local inflammatory response lies in the presence of silicone-derived products or specific bacterial species adherent to the prosthesis surface (biofilm) [20, 22]. The median interval time between breast implant surgery and BIA-ALCL diagnosis is 9 years [13]. Thus, the implant must be in place for a long time before the pathology appears. This adds an additional explanation to the development of BIA-ALCL. The irritation caused by the textured implant occurs long after the surgery and the development of the pathology. Some cases of BIA-ALCL with smooth-implant have been reported, but no details concerning previous surgery with breast implant silicone have been listed. The FDA confirmed that BIA-ALCL is predominantly associated with textured surface implants [8].

Therefore, a systematic description of each implant, ideally the reference/manufacturer, and a list of all surgeries made on the patient must be reported for new cases. A lot of studies report BIA-ALCL without disruption of the prosthesis like our two cases, suggesting that there is no influence on the development of this pathology [9].

Moreover, the increasing number of breast prosthesis implanted could result in an increased of diagnoses of breast implant-associated ALCL thus encouraging clinical and radiological surveillance of these patients. Radiologists and senologists must be particularly attentive when they observe a rapid increase in breast volume of patients with breast prosthesis. In November 2018, the superior health council of Belgium also provided recommendations for the detection, registration, and follow-up of BIA-ALCL. In their report, they recommend plastic surgeons include an informed consent about the risk of developing BIA-ALCL for patients having an augmentation or breast reconstruction using implants [23].

In conclusion, our study reinforce the need to report each case of BIA-ALCL to better understand this rare pathology and demonstrates the use of a new approach to highlight periprosthetic effusion or modification of the prosthesis. Mammography without compression allows us to correctly locate the prosthesis (retropectoral or retroglandular) and search for disruption of the prosthesis or liquid collection. When BIA-ALCL is suspected on mammography, it is also recommended to perform an echography and a fine needle aspiration of seroma followed by cytological evaluation and CD30 immunohistochemistry.

## Supplementary Materials

Supplementary Materials This figure demonstrates a CD30 stain performed on the capsules for each case. CD30 immunohistochemistry remains negative. (a) Case 1; (b) Case 2.

## References

[1] Talwalkar S. S., Miranda R. N., Valbuena J. R., Routbort M. J., Martin A. W., Medeiros L. J. (2008). Lymphomas involving the breast: a study of 106 cases comparing localized and disseminated neoplasms. *The American Journal of Surgical Pathology*.

[2] Giardini R., Piccolo C., Rilke F. (1992). Primary Non‐Hodgkin's lymphomas of the female breast. *Cancer*.

[3] Validire P., Capovilla M., Asselain B. (2009). Primary breast non-Hodgkin's lymphoma: a large single center study of initial characteristics, natural history, and prognostic factors. *American Journal of Hematology*.

[4] Gualco G., Chioato L., Harrington W. J., Weiss L. M., Bacchi C. E. (2009). Primary and secondary T-cell lymphomas of the breast: clinico-pathologic features of 11 cases. *Applied Immunohistochemistry & Molecular Morphology *.

[5] Lazzeri D., Agostini T., Bocci G. (2011). ALK-1–Negative Anaplastic Large Cell Lymphoma Associated With Breast Implants: A New Clinical Entity. *Clinical Breast Cancer*.

[6] Keech J. A., Creech B. J. (1997). Anaplastic T-cell lymphoma in proximity to a saline-filled breast implant. *Plastic and Reconstructive Surgery*.

[7] Feldman A. L., Harris N. L., Stein H., Swerdlow S. H., Campo E., Harris N. L. (2017). Breast implant-associated anaplastic large cell lymphoma. *WHO classification of tumours of haematopoietic and lymphoid tissues*.

[8] Clemens M. W. BIA-ALCL Ressources. By the numbers, and what they meen. ASPS Spring meeting. https://www.plasticsurgery.org/for-medical-professionals/health-policy/bia-alcl-physician-resources/by-the-numbers.

[9] Laurent C., Delas A., Gaulard P. (2016). Breast implant- associated anaplastic large cell lymphoma: two distinct clinicopathological variants with different outcomes. *Annals of Oncology*.

[10] Cezkwudo D., Ifabiyi T., Gbadamosi B. (2017). Breast implant-associated anaplastic large cell lymphoma: a case report and review of the literature. *Case Reports in Oncological Medicine*.

[11] Clemens M. W., Medeiros L. J., Butler C. E. (2016). Complete surgical excision is essential for the management of patients with breast implant–associated anaplastic large-cell lymphoma. *Journal of Clinical Oncology*.

[12] Mehta-Shah N., Clemens M. W., Horwitz S. M. (2018). How I treat breast implant–associated anaplastic large cell lymphoma. *Blood*.

[13] Quesada A. E., Medeiros L. J., Clemens M. W. (2019). Breast implant-associated anaplastic large cell lymphoma: a review. *Modern Pathology*.

[14] Eklund G., Busby R., Miller S., Job J. (1988). Improved imaging of the augmented breast. *American Journal of Roentgenology*.

[15] Letter H., Rop B., Edison M. N., Turner P. (2016). Breast implant-associated anaplastic large cell lymphoma: a case report and literature review. *Cureus*.

[16] Rupani A., Frame J. D., Kamel D. (2015). Lymphomas associated with breast implants: a review of the literature. *Aesthetic Surgery Journal*.

[17] Clemens M., Miranda R. (2015). Coming of age breast implant-associated anplastic large cell lymphoma after 18 years of investigation. *Clinics in Plastic Surgery*.

[18] Clemens M. W., Medeiros L. J., Butler C. E. (2016). Surgical excision is essential for the management of patients with breast implant-associated anaplastic large-cell lymphoma. *Journal of Clinical Oncology*.

[19] Clemens M. W., Brody G. S., Mahabir R. C., Miranda R. N. (2018). How to diagnose and treat breast implant–associated anaplastic large cell lymphoma. *Plastic and Reconstructive Surgery*.

[20] de Boer M., van Leeuwen F. E., Hauptmann M. (2018). Breast implants and the risk of anaplastic large-cell lymphoma in the breast. *JAMA Oncology*.

[21] Brody G. S., Deapen D., Taylor C. R. (2015). Anaplastic large cell lymphoma occurring in women with breast implants. *Plastic and Reconstructive Surgery*.

[22] Hu H., Johani K., Almatroudi A. (2016). Bacterial biofilm infection detected in breast implant–associated anaplastic large-cell lymphoma. *Plastic and Reconstructive Surgery*.

[23] Federal Public Service Health Advisory report 9473 – Breast Implant. https://www.health.belgium.be/en/advisory-report-9473-breast-implant.

