# Supplementary figures and images for: Description of Two Cases of Anaplastic Large Cell Lymphoma Associated with a Breast Implant
Source: Case Rep Radiol. 2019 Jun 27;2019:6137198. doi: 10.1155/2019/6137198 (PMC6620858; doi:10.1155/2019/6137198)

**Supplementary figure: CD30 immunochemistry is negative in the lining (original magnification 50X).**

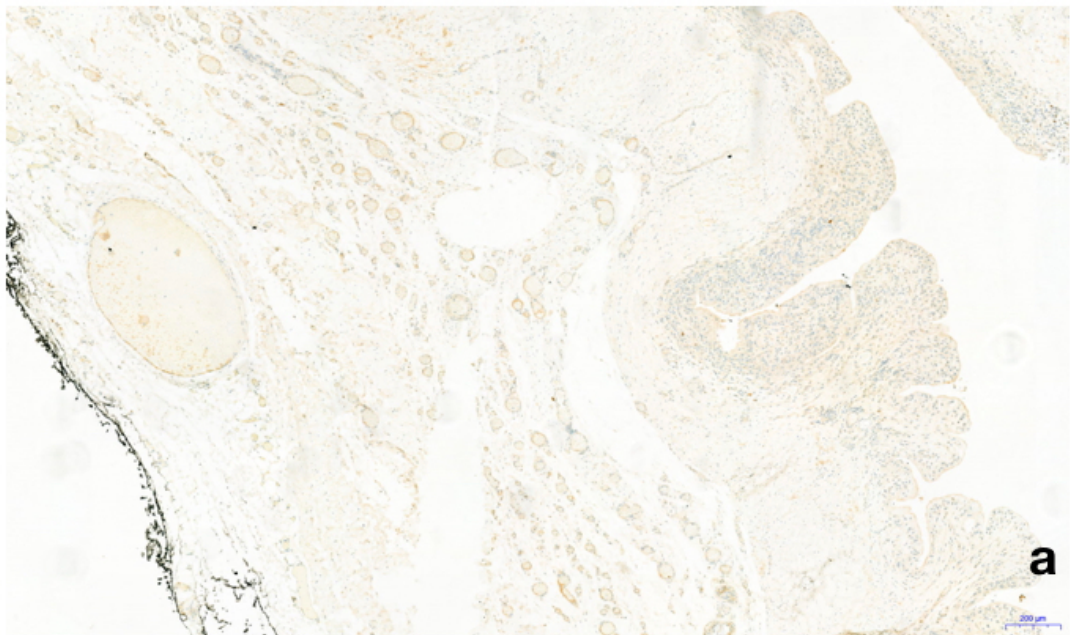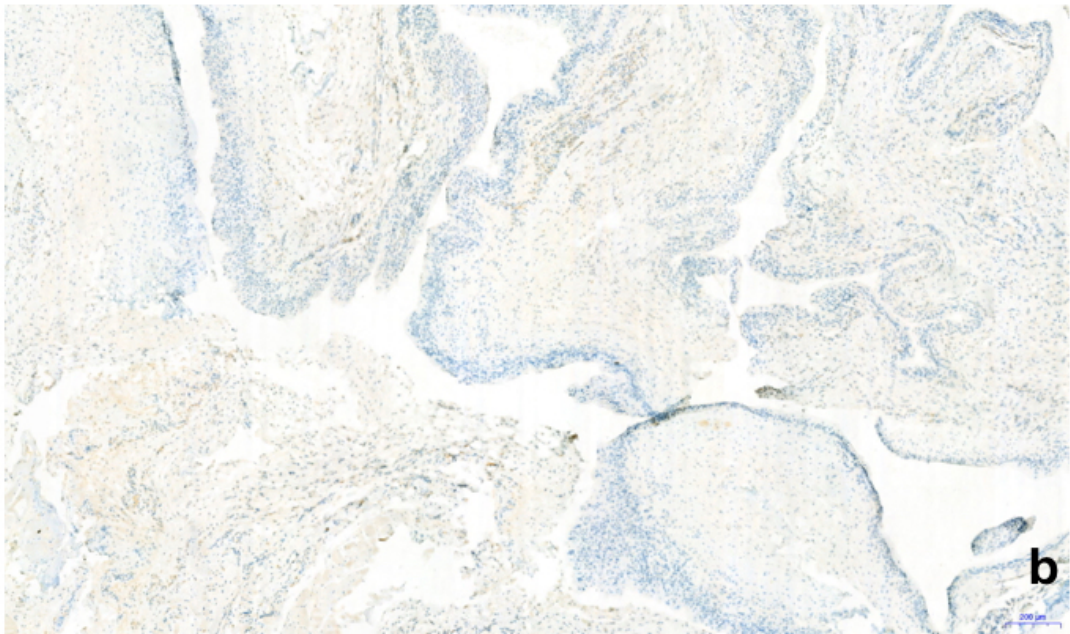

Supplement: Supplementary Materials — This figure demonstrates a CD30 stain performed on the capsules for each case. CD30 immunohistochemistry remains negative. (a) Case 1; (b) Case 2. [file 6137198.f1.pdf]
